# Infant feeding knowledge among women living with HIV and their interaction with healthcare providers in a high-income setting: a longitudinal mixed methods study

**DOI:** 10.1186/s13006-024-00677-2

**Published:** 2024-10-11

**Authors:** Ellen Moseholm, Inka Aho, Åsa Mellgren, Isik S Johansen, Terese L Katzenstein, Gitte Pedersen, Merete Storgaard, Nina Weis

**Affiliations:** 1 Department of Infectious Diseases, Copenhagen University Hospital, Kettegaard Alle 30, Hvidovre, Denmark; 2 Department of Public Health, Faculty of Health and Medical Sciences, University of Copenhagen, Copenhagen, Denmark; 3 Department of Infectious Diseases, Helsinki University Hospital, Helsinki, Finland; 4 Department of Infectious Diseases, Region Västra Götaland, Sahlgrenska University Hospital, Gothenburg, Sweden; 5 Department of Infectious Diseases, Institute of Biomedicine, Sahlgrenska Academy, University of Gothenburg, Gothenburg, Sweden; 6 Department of Infectious Diseases, Odense University Hospital, Odense, Denmark; 7 Department of Infectious Diseases, Copenhagen University Hospital, Rigshospitalet, Copenhagen Ø, Denmark; 8 Department of Infectious Diseases, Aalborg University Hospital, Aalborg, Denmark; 9 Department of Infectious Diseases, Aarhus University Hospital, Aarhus, Denmark; 10 Department of Clinical Medicine, Faculty of Health and Medical Sciences, University of Copenhagen, Copenhagen, Denmark

**Keywords:** Women living with HIV, Infant feeding, Breastfeeding, Longitudinal-mixed methods study, Nordic setting, 2BMOM

## Abstract

**Background:**

Recent changes in the infant feeding guidelines for women living with HIV from high-income countries recommend a more supportive approach focusing on shared decision-making. Limited information is available on the infant feeding knowledge of women living with HIV and how healthcare providers engage with them in this context. This multicenter, longitudinal, mixed methods study aims to get a comprehensive and nuanced understanding of infant feeding knowledge among women living with HIV of Nordic and non-Nordic origin living in Nordic countries, and their interaction with healthcare providers regarding infant feeding planning.

**Methods:**

Pregnant women living with HIV in Denmark, Finland, and Sweden were recruited in 2019–2020. The Positive Attitudes Concerning Infant Feeding (PACIFY) questionnaire was completed in the 3rd trimester (T1), three (T2), and six (T3) months postpartum. Women who completed the quantitative survey were also invited to participate in qualitative semi-structured interviews at T1 and T3. Results from the survey and interviews were brought together through merging to assess for concordance, complementarity, expansion, or discordance between the datasets and to draw meta-inferences.

**Results:**

In total, 44 women living with HIV completed the survey, of whom 31 also participated in the interviews. The merged analyses identified two overarching domains: Knowledge about breastfeeding in the U = U era and Communications with healthcare providers. The women expressed confusion about breastfeeding in the context of undetectable equals untransmittable (U = U). Women of Nordic origin were more unsure about whether breastfeeding was possible in the context of U = U than women of non-Nordic origin. Increased postpartum monitoring with monthly testing of the mother was not seen as a barrier to breastfeeding, but concerns were found regarding infant testing and infant ART exposure. Infant feeding discussions with healthcare providers were welcome but could also question whether breastfeeding was feasible, and many participants highlighted a need for more information.

**Conclusions:**

Healthcare providers caring for women living with HIV must have up-to-date knowledge of HIV transmission risks during breastfeeding and engage in shared decision-making to optimally support infant feeding choices.

## Background

The success of combination antiretroviral therapy (ART) has reduced perinatal HIV transmission in many parts of the world, including the Nordic countries, to less than 1% [1–3]. Perinatal HIV transmission refers to the transmission of HIV from mother to child during pregnancy, delivery, or postpartum via breastfeeding. The estimated risk of HIV transmission through breastfeeding without maternal ART is 15-30% over a two-year period [4]. Results from the Promoting Maternal and Infant Survival Everywhere (PROMISE) trial showed that with successful maternal ART during pregnancy and postpartum the transmission risk during breastfeeding decreases to less than 1% [5, 6]. However, both the PROMISE trial [5, 6] and other transmission studies [7, 8] were conducted in low- and middle-income countries and most postnatal transmissions occurred in women who initiated ART late in pregnancy or among women with adherence challenges and/or detectable viremia. There is limited evidence on the transmission risk through breastfeeding in women who have been treated with ART throughout the whole pregnancy and postpartum period.

In most high-income countries (including the Nordic countries) where access to safe and accessible infant feeding options are readily accessible, exclusive formula feeding is recommended as the safest option for women living with HIV (WLWH) [9–12]. Qualitative studies have highlighted that some WLWH, especially those originating from low- and middle-income settings where breastfeeding is recommended irrespective of HIV status [13], may face personal, social, and familial pressures to breastfeed [14–16]. Fear of others finding out about HIV status has also been described as a concern [17]. Moreover, following the results of the PARTNER study, confirming that individuals with an undetectable viral load do not transmit HIV sexually [18], referred to as undetectable equals untransmittable (U = U), there has been much debate in the literature on whether this also applies to breastfeeding, particularly for women on ART throughout the pregnancy and the postpartum period [19–22].

Experts and patients have in the past decade called for a shared decision-making approach, where WLWH receive the information and support necessary to make informed infant feeding decisions [22–25]. This has led to recent updates in guidelines emphasizing that counseling on infant feeding is an integral component of care for pregnant and postpartum WLWH and that WLWH should be supported in their choice of infant feeding, whether this is formula feeding or breastfeeding [11, 12].

Many factors influence infant feeding choices, including social and cultural factors, personal values, desire for infant bonding, and stigma [20, 26] and WLWH in high-income countries are increasingly choosing to breastfeed [26–28]. Healthcare providers (HCPs) play an important role in supporting safe infant feeding choices in the context of HIV. Recent studies from the US have documented that HCPs are being asked about breastfeeding in the context of HIV and that providers often have limited experience and knowledge when counseling WLWH on infant feeding choices [29, 30]. However, studies have also shown that many WLWH either do not receive counseling or are unsatisfied with the infant feeding counseling they do receive, and may not fully understand the scenarios where breastfeeding could be supported (e.g., in the context of fully suppressed HIV viral load) [31, 32]. Thus, guidance from HCPs and knowledge about infant feeding choices is an important component of care for pregnant and postpartum WLWH and the shared decision making process [33]. However, as highlighted in a recent meta-synthesis, there is a scarcity of research on infant feeding knowledge and counseling among WLWH living in high-income settings, especially in the context of U = U [14].

Using a mixed methods research design this study aimed to get a comprehensive and nuanced understanding of infant feeding knowledge among WLWH living in Nordic countries, their interaction with and support by HCPs regarding infant feeding choices, and to assess differences between WLWH of non-Nordic and Nordic origin.

## Methods

### Design

**Fig. 1:**
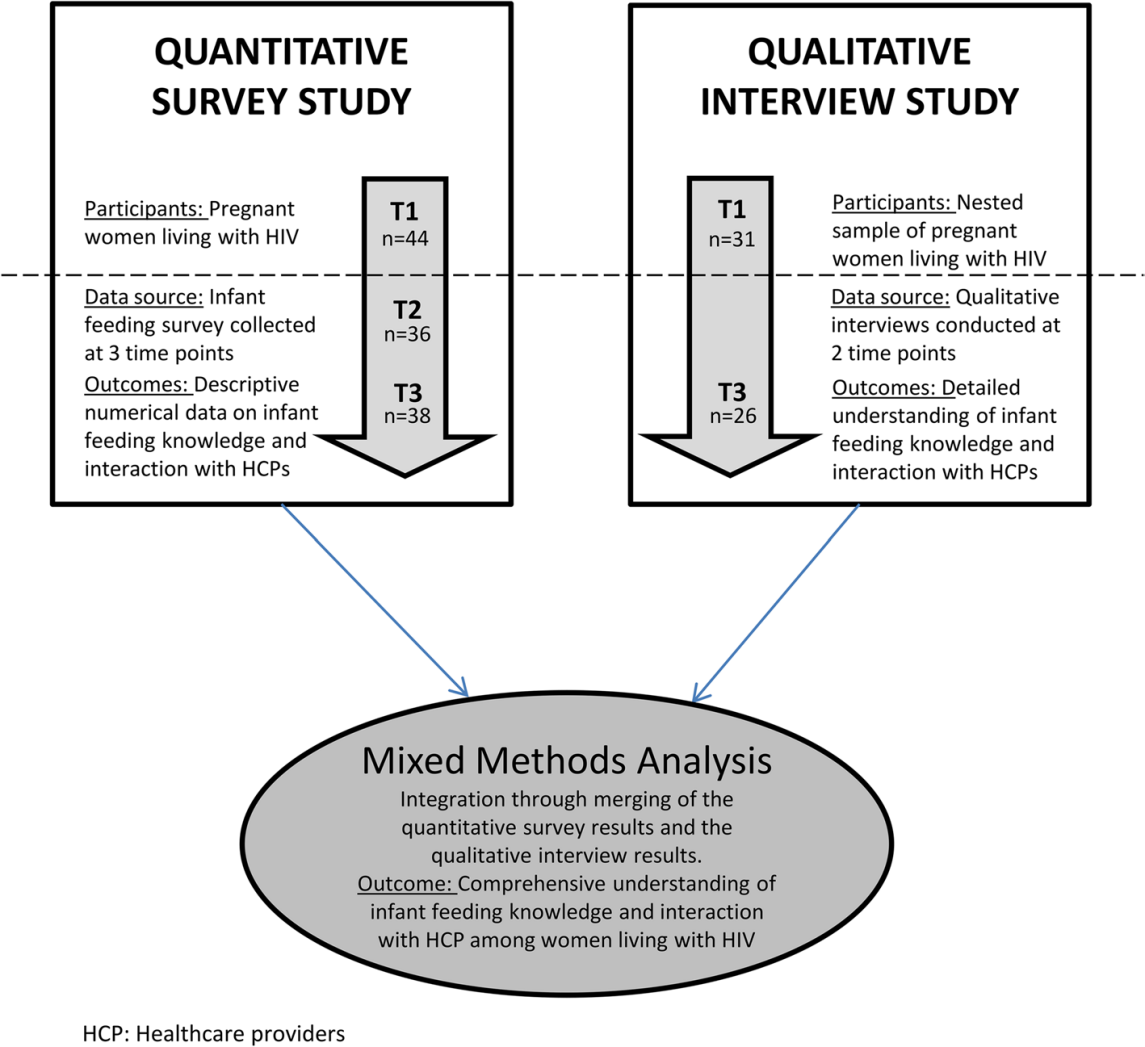
Study diagram

 This study used data from the “Becoming and Being a Mother Living with HIV” (2BMOM) study, a multi-center, longitudinal, convergent mixed methods study among pregnant and postpartum WLWH in the Nordic countries Denmark, Finland, and Sweden [34]. The 2BMOM study, which is described in detail elsewhere [34], consisted of a survey study [35] and a qualitative interview study [36] (Fig. 1). Using multiple methods describing both general trends and detailed in-depth data on infant feeding knowledge and experiences with HCPs can enhance understanding to guide support regarding infant feeding choices among WLWH [37].

## Setting

There are approximately 5,400 WLWH living in Denmark, Finland, and Sweden [38–40]; the majority of whom have immigrated mainly from sub-Saharan Africa. The tax-based healthcare system in the Nordic countries ensures universal access to both medical healthcare and many social support services [41]. Thus, ART is provided free of charge, and people living with HIV are generally well-treated on ART with life expectancies approaching those of the general population [39, 40, 42]. Most pregnant WLWH in the Nordic countries have an undetectable viral load at the time of delivery resulting in a perinatal HIV transmission rate of < 1% [1, 2, 43]. Guidelines in the participating countries do not recommend breastfeeding for WLWH [9, 44, 45]. However, if a woman with an undetectable viral load decides to breastfeed, she should be supported in this choice and there should be increased monitoring of both mother and child throughout the breastfeeding period [9, 44].

## Quantitative survey

### Participants

Pregnant WLWH were consecutively recruited by the medical staff during routine clinical appointments between January 2019 and December 2020 from the participating sites: Departments of Infectious Diseases at Copenhagen University Hospitals, Hvidovre, and Rigshospitalet; Odense -, Aalborg – and Aarhus University Hospitals in Denmark, Helsinki University Hospital, Finland, and Sahlgrenska University Hospital, Sweden. Women were eligible for inclusion if they were ≥ 18 years of age, living with HIV, pregnant with a viable infant without life-threatening conditions or congenital anomalies, and able to speak and read English, Danish, Swedish, or Finnish.

## Data collection

Quantitative data were collected using self-administered questionnaires via REDCap© in the 3rd trimester (T1), three and six months postpartum (T2 and T3, respectively). At each time point, a survey link was sent to the participants, who then completed the survey on their own time. Infant feeding knowledge was assessed using the Positive Attitudes Concerning Infant Feeding (PACIFY) questionnaire, which contains 20 items assessing knowledge and different views and issues surrounding breastfeeding in WLWH [31]. The questionnaire was translated into Danish, Finnish, and Swedish using the forward-backward method [46]. Demographic variables were collected at baseline (T1). Information on clinical variables were obtained from the medical records.

### Analysis

For comparison, descriptive data were stratified by migration status defined as non-Nordic origin (born outside a Nordic country) and Nordic origin (born in a Nordic country). Categorical variables were described as counts and calculated percentages, and continuous variables were described as means (95% confidence intervals (CI)). Analyses were performed using STATA 17 software.

## Qualitative interviews

### Participants

Eligible participants included pregnant WLWH who had completed the survey at T1 and who could speak Danish or English. Thus, a nested sample of WLWH were recruited for both the survey and the interviews at the time of recruitment into the study. Participants were consecutively sampled until reaching data saturation (i.e. the point when no substantially new information emerged from the interviews) [47, 48].

### Data collection

Qualitative data were collected via individual interviews conducted by the first author (EM) in the third trimester (T1) and six months postpartum (T3). All interviews used a hybrid, narrative/semi-structured format [49]. This approach was chosen to ensure that the same concepts were explored in both the quantitative and qualitative phases of the 2BMOM study, while still allowing for new insights and perspectives to emerge [34]. The interviews were conducted in Danish or English in the home of the participant, at the relevant hospital, or online using a video meeting setup, based on the women’s preference. The interviews lasted between 20 and 90 min (mean 51 min), were audio-recorded, and transcribed verbatim.

### Analysis

The transcribed interviews were analyzed inductively using narrative thematic analysis as described by Riessman [50]. The analysis consisted of several consecutive steps: (1) Initial coding focusing on capturing the main ideas from the women´s stories. (2) Emergent themes and patterns across a subset of transcripts were identified and discussed among the team members (EM, NW) while paying close attention to the whole story and the study aims. (3) The themes were then compared for similarities and differences across participants and their narratives, focusing on women of non-Nordic and Nordic origin, respectively. (4) The themes were brought together to create and define the primary narrative themes [50]. (5) Consistency across the themes was discussed and a codebook was developed to document and organize the codes. In the final step, the codebook was used to code and analyze all the interview data using NVivo software, ©QSR International Pty Ltd.

### Mixed methods integration and analysis

Integration in mixed methods research is defined as an intentional process by which the researcher brings qualitative and quantitative data together in one study [47]. The quantitative survey data and qualitative interview data in this study were brought together through merging using identified commonalities across the two datasets as the overarching domains [51]. Specifically, the results from the two datasets were merged in a joint display analysis using a side-by-side comparison to assess for complementarity, expansion, concordance, or discordance between the datasets and to draw meta-inferences (i.e., interpretations made based on both the qualitative and quantitative findings) [51–53] for WLWH of non-Nordic and Nordic origin. Complementarity occurred when the two datasets illustrated different but nonconflicting interpretations. Expansion occurred when the findings from the two datasets diverged and expanded insights by addressing different aspects of infant feeding knowledge and perceptions. Concordance occurred if the findings from both types of data led to the same interpretation, while discordance occurred if the survey and interview results were contradictory or disagreed with each other [52]. The results are presented in joint displays where the quantitative and qualitative results are visualized side-by-side in a table together with the meta-inferences [51, 53].

## Results

### Participant characteristics

Overall, 71 pregnant WLWH fulfilled the inclusion criteria during the study period of whom 57 agreed to participate in the quantitative survey and 47 pregnant women completed the baseline survey (T1). The main reasons for non-participation were language barriers and psychiatric or social complications. The PACIFY questionnaire was completed by 44 women at T1 and were thus included in this analysis (response rate 62%). A total of 36 and 38 women (82% and 86%) completed the survey at follow-up T2 and T3, respectively (Fig. 1). In total, 31 pregnant WLWH agreed to participate in the qualitative interviews including 24 from Denmark, five from Finland, and two from Sweden. Baseline characteristics are presented in Table 1. All participating women identified as cisgender. Twelve women were born in a Nordic country, while 32 were born outside of the Nordic countries; 24 of whom were of African origin, while eight originated from South America, Southern or Eastern Europe, or the Middle East. All women were on ART at the time of delivery.

**Table 1:** Baseline characteristics of women living with HIV included in the study

	Quantitative Survey	Qualitative Interviews
	(*n* = 44)	(*n* = 31)
**Age**,** mean (95% CI)**	33.91 (32.4 : 35.5)	33.9 (29.5 : 36.6)
**Relationship status**,** n (%)**		
Married/living with a partner	35 (80)	25 (80)
Have a partner, but not living together	4 (9)	3 (10)
Do not have a current partner	5 (11)	3 (10)
**Country of birth**,** n (%)**		
Nordic country (Denmark, Finland or Sweden)	12 (27)	9 (29)
Africa	24 (55)	19 (61)
Other	8 (18)	3 (10)
**Education**,** n (%)**		
Primary/Secondary school	14 (32)	11 (35)
Higher education (college/university)	27 (61)	20 (65)
Unknown	3 (7)	0
**Nulliparous**,** n (%)**	15 (34)	14 (45)
**Years since HIV diagnosis**,** mean (95% CI)**	9.32 (7.0 ; 11.6)	9.55 (6.4 ; 12.7)
**HIV diagnosis in pregnancy**,** n (%)**		
Yes	3 (7)	3 (10)
**Mode of HIV transmission**,** n (%)**		
Sexual	39 (89)	27 (87)
Perinatal	5 (11)	4 (13)
HIV viral load*,** n (%)**		
< 50 copies/mL	36 (82)	24 (77)
>=50 copies/ml	8 (18)	7 (23)
**Mode of delivery**,** n (%)**		
Vaginal	31 (70)	22 (71)
Caesarean	13 (30)	9 (29)
**Gestational age < 37 weeks**,** n (%)**	4 (9)	< 3 (6)

* At baseline (T1). All participants had an HIV RNA viral load < 50 at the time of delivery.

### Mixed methods analysis

The merged analyses identified two overarching domains: Knowledge about breastfeeding in the U = U era and Communications with healthcare providers.

### Knowledge about breastfeeding in the U = U era

The results on knowledge about breastfeeding in the U = U era are presented in Table 2. In the survey, 75% of participants responded that it was not safe to breastfeed with a detectable HIV viral load, irrespective of maternal origin and with little change over time (non-Nordic origin *n* = 24/32 and Nordic origin *n* = 9/12 at T1). Half of the women of non-Nordic origin responded at T1 either that it was safe to breastfeed with an undetectable HIV viral load (*n* = 7/32) or that they did not know (*n* = 8/32). Half of the women of Nordic origin responded at T1 that it was safe to breastfeed with an undetectable HIV viral load (*n* = 6/12), while one-third responded that they did not know (*n* = 4/12). At T2 and T3, 75% (*n* = 9/12) and 60% (*n* = 6/10) of the WLWH of Nordic origin responded that they did not know whether it was safe to breastfeed with an undetectable viral load.

**Table 2:**
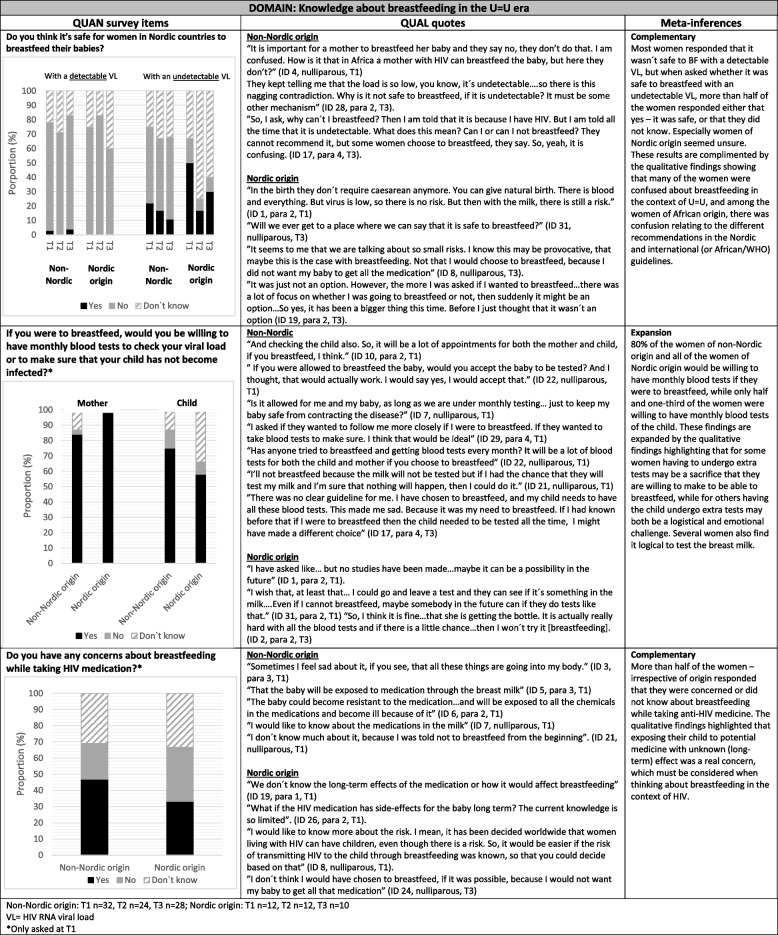
Knowledge about breastfeeding in the U=U era among women living with HIV of non-Nordic and Nordic origin depicted in a joint display of quantitative, qualitative, and mixed methods findings

The qualitative results complemented these findings showing that many of the women were confused about breastfeeding in a U = U context. Several of the women questioned why there was a risk of HIV transmission through breastfeeding when they could not transmit HIV through sex if they had an undetectable viral load. The women also noted that they could have a vaginal delivery, even though the risk of HIV transmission could not be completely ruled out, and wondered why this was not also the case with breastfeeding. Women who immigrated, especially women of African origin, found it confusing that the guidelines in their home country recommended breastfeeding in WLWH, while in the Nordic countries, breastfeeding was not recommended.

All of the women of Nordic origin (*n* = 12/12) and most of the women of non-Nordic origin (*n* = 27/32) would be willing to have monthly blood tests to check viral load if they were to breastfeed. However, participants were less likely to have additional blood tests taken on their child (non-Nordic origin *n* = 24/32 and Nordic origin *n* = 7/12). The qualitative findings revealed that women saw additional tests as a practical way to make breastfeeding with HIV safer, providing reassurance for some. However, the women were also concerned about the number of appointments this would require and putting their child through additional blood tests. One mother decided to breastfeed but was unprepared for the additional monthly tests her child required, leading her to stop breastfeeding after two months. 47% (*n* = 15/32) of women of non-Nordic origin and 33% (*n* = 4/12) of women of Nordic origin responded that they had concerns about breastfeeding while on ART, while one-third in both groups responded that they did not know. The qualitative findings highlighted that exposure to antiretroviral (ARV) drugs through breastfeeding was something the women considered when balancing between the risks and benefits of breastfeeding. A few women stated that this was not something they had thought about, because breastfeeding was not an option when living with HIV.

### Communications with healthcare providers

The results on communications with HCPs are presented in Table 3. A majority of the women in both groups had discussed breastfeeding with their HCPs; 84% (*n* = 27/32) at T1, 88% (21/24) at T2 and 89% (*n* = 25/28) at T3 among women of non-Nordic origin and 83% (*n* = 10/12) at T1, 75% (*n* = 9/12) at T2, and 80% (*n* = 8/10) at T3 among women of Nordic origin. The qualitative findings expanded this showing that discussions with HCPs ranged from hardline recommendation of breastfeeding avoidance to more open-ended questions about infant feeding/breastfeeding. These open-ended questions and discussions were welcome but could also add to the confusion about whether breastfeeding was possible. Some women brought up the subject of infant feeding themselves, seeking information, especially with what to say if asked about breastfeeding by others.

**Table 3:**
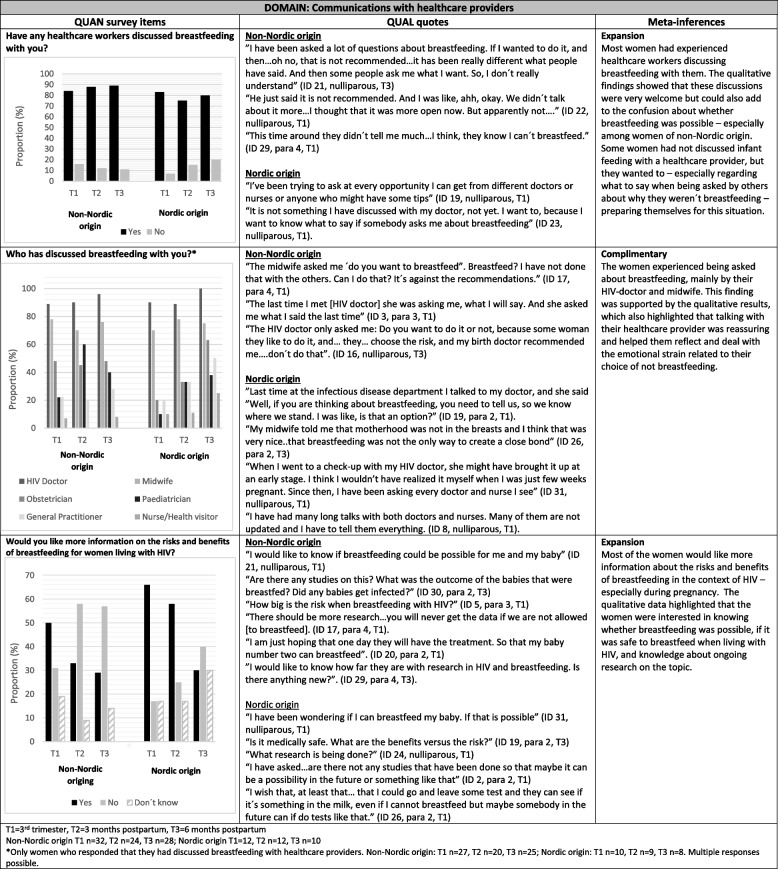
Communications with healthcare providers among women living with HIV of non-Nordic and Nordic origin depicted in a joint display of quantitative, qualitative, and mixed methods findings

The women had mainly discussed breastfeeding with their HIV doctor and their midwife; 89% (*n* = 24/27) and 78% (*n* = 21/20) of women of non-Nordic origin and 90% (*n* = 9/10) and 70% (*n* = 7/10) of women of Nordic origin, respectively. However, the quantitative results also highlight that the women discussed breastfeeding with a wide range of HCPs across the pregnancy-postpartum trajectory. These discussions were often initiated by the HCPs. The qualitative results complimented these findings showing that these discussions were experienced as reassuring by many of the women, helping them reflect and deal with the emotional strain related to their choice of not breastfeeding. However, the qualitative findings also revealed that the women often felt healthcare providers lacked up-to-date knowledge about HIV, particularly in the context of infant feeding, leading them to feel they had to educate the providers.

Half of the women of non-Nordic origin (*n* = 16/32) and 66% (*n* = 8/12) of the women of Nordic origin would like more information on the risks and benefits of breastfeeding for WLWH in pregnancy. The need for information was less in the postpartum period, especially among the women of non-Nordic origin. The qualitative data expanded on these findings highlighting that the women were interested in knowing whether breastfeeding was possible, whether it was safe to breastfeed while living with HIV, and knowledge about what research was being done in the area.

## Discussion

The findings from this mixed methods study on infant feeding knowledge among WLWH in Nordic countries and their interaction with HCPs found that there was confusion about breastfeeding in the context of U = U, that the women did discuss infant feeding with HCPs across the pregnancy and postpartum trajectory, and that these discussions were welcome, but could also add to the confusion about whether breastfeeding was feasible.

Several of the previous studies exploring infant feeding knowledge have been conducted prior to the introduction of U = U with a focus on WLWH who have migrated to either the UK or the USA [16, 54, 55]. More recent qualitative studies conducted among WLWH in Canada and the USA [56, 57] support our finding that the recommendation of breastfeeding avoidance in a U = U context is difficult to understand for many WLWH. Our finding about confusion related to the difference in infant feeding guidelines, especially among women who have migrated from low- and middle-income countries where breastfeeding is recommended irrespective of HIV status, have been reported in previous studies [16, 54, 57]. However, the quantitative findings from our study show that women of Nordic origin seem to be more unsure about the safety of breastfeeding with an undetectable viral load compared to women of non-Nordic origin. In the PACIFY study, one-third of 94 WLWH in the UK responded that they did not know if it was safe to breastfeed with an undetectable viral load [31]. Knowledge regarding HIV transmission has been shown to provide confidence in infant feeding choices in WLWH while varying advice from HCPs and difference in guidelines can create ambiguity and insecurity about HIV transmission risks [58]. Although women in both groups would like more information about the risks and benefits of breastfeeding when living with HIV, especially during pregnancy, the need for more information was highest among women of Nordic origin.

International guidelines for pregnant and postpartum women with HIV recommend increased monitoring of both mother and child throughout the breastfeeding period [11, 12]. Almost all WLWH in our study would agree to have additional blood tests drawn for themselves in case of breastfeeding. However, approximately a quarter of WLWH of non-Nordic origin and one-third of WLWH of Nordic origin did not want or were unsure about additional testing of their child. Infant HIV testing has been described as emotionally difficult and associated with feelings of guilt and sadness by other studies [15–17, 54]. Our results highlight that preparing WLWH who choose to breastfeed about the additional testing of the child is important, as this may influence their infant feeding choice. Another important aspect is infant ARV exposure through breast milk. ARVs are passed through breast milk, although the clinical relevance of ARV concentrations in breast milk is not fully understood [59]. Serious adverse events in infants, due to maternal ART, appear to be uncommon [20]. However, our results show that ARV exposure through breast milk is relevant to WLWH when considering their infant feeding choices.

The majority of WLWH had discussed infant feeding with their HCP, a finding that is supported by the PACIFY study [31]. What this study adds is knowledge about the quality and debts of these conversations.

Our findings highlight that the women want to engage in discussions about infant feeding. This is supported by a recent US study [15]. Our results also highlight that the discussions about infant feeding may add to the confusion about whether breastfeeding is possible in a U = U context. Several women experienced that the discussions with HCPs were limited to a recommendation of breastfeeding avoidance. Rather than focusing on breastfeeding or not, WLWH emphasizes a more comprehensive perspective of choice in relation to infant feeding [17, 57]. Infant feeding discussions among women who chose not to breastfeed were experienced as supportive and reassuring, especially with concerns about bonding. Moreover, receiving help with developing strategies on what to say when asked about breastfeeding was also important for many participants, a finding supported by others [29, 56].

### Clinical implications

Implementing a shared decision-making approach to support infant feeding choices can help WLWH to understand the risk of transmission with breastfeeding and why U = U does not, with the current knowledge, apply to breastfeeding, and also accept global differences in guidelines [57, 60]. This requires that the risks and benefits of breastfeeding in the context of HIV are discussed, in addition to frequent follow-up visits for both the mother and infant if the mother decides to breastfeed [60].

Advising in the context of many unanswered questions and distinct lack of evidence may be challenging for many HCPs [30, 61]. Findings from a recent US study show that HCPs struggle with the tension between responding to patients´ choices, while simultaneously protecting infants from risk of infection and following official guideline recommendations [30]. Examples of how to discuss infant feeding with WLWH have been published [61, 62]. What these have in common is that counseling should be ongoing throughout the pregnancy and postpartum period, that HCPs should be honest about the lack of evidence, and informing the women that the best way to eliminate risk is to abstain from breastfeeding [61, 62].

Infant feeding is a social, cultural, and emotional issue that is best understood in relationship to the women’s cultural and social background, and as WLWH [15, 17]. Thus, it is important that HCPs actively listen and answer questions without judgment [56] and takes into account how culture and HIV-related stigma intersect with infant feeding knowledge and experiences when engaging in shared decision-making [17]. Initiating an open and honest conversation about infant feeding options based on current evidence and guidelines is crucial and resources from national HIV organizations and local NGO´s are available to support HCP during this process [63–66].

### Strengths and limitations

To our knowledge, this is the first study focusing specifically on infant feeding knowledge and experiences in WLWH living in Nordic countries. Using multiple methods provided a more comprehensive and nuanced understanding of infant feeding knowledge and experience with HCP. The small sample size and the risk of selection bias is a limitation, reducing generalizability. The number of pregnant WLWH in the Nordic countries are small (< 70/year at the participating sites) and although > 60% of eligible women were included, this study does not reflect the perspective of WLWH who do not speak a native Nordic language or English.

The term “breastfeeding” was used in the survey. Although we acknowledge that there are multiple terms, including chestfeeding used to describe this process, the results cannot necessarily be extrapolated to apply to chestfeeding. Moreover, we used a hybrid, narrative/semi-structured format in the interviews to ensure that overarching themes were explored in both the quantitative and qualitative strands. Although in conformity with the aim of the overall study, this approach may have limited the elaboration of the women´s experiences. Finally, the study was completed during the COVID-19 pandemic, which could have had an impact on the results. The participants may have had less contact with HCPs and other support systems during this time, potentially impacting the amount of information they received about infant feeding.

## Conclusion

This mixed methods study among WLWH with different backgrounds living in a high-income setting highlights that WLWH are confused about breastfeeding choices and transmission risk. Women of Nordic origin were more unsure about whether breastfeeding was possible in the context of U = U than women of non-Nordic origin. The study also found that increased postpartum monitoring with monthly testing of the mother was not seen as a barrier to breastfeeding, but concerns were found regarding infant testing and infant ART exposure. Infant feeding discussions with HCPs across the pregnancy and postpartum trajectory were welcome, irrespective of origin. Thus, HCPs caring for WLWH must have updated knowledge about HIV transmission risk during breastfeeding and initiate a shared decision-making process to provide optimal support for infant feeding choices in WLWH.

## Data Availability

The data that support the findings of this study are available from the corresponding author, but restrictions apply to the availability of these data, which were used under license for the current study, and so are not publicly available. Data are however available from the authors upon reasonable request and with permission of relevant regulatory agencies.

## Abbreviations

ART: Antiretroviral Therapy
ARV: Antiretroviral
HCP: Healthcare providers
PACIFY: Positive Attitudes Concerning Infant Feeding
The 2BMOM study: The Becoming and Being a Mother living with HIV study
WLWH: Women Living with HIV
U = U: Undetectable equals untransmittable
